# Molecular screening of tick-borne pathogens in host-seeking *Haemaphysalis punctata* Canestrini & Fanzago, 1878 (Ixodoidea: Ixodidae) in Anatolia with the first report of Burana virus

**DOI:** 10.1186/s13071-026-07367-4

**Published:** 2026-03-27

**Authors:** Ömer Orkun, Tuğba Özdemir, Maide Nur Gündoğdu, Mesut Yiğit, Ahmet Deniz, Zati Vatansever

**Affiliations:** 1 Ticks and Tick-Borne Diseases Research Laboratory, Department of Parasitology, Faculty of Veterinary Medicine, Ankara University, 06070 Ankara, Türkiye; 2 Graduate School of Health Sciences, Ankara University, Ankara, Türkiye; 3 Department of Parasitology, Faculty of Veterinary Medicine, Kafkas University, Kars, Türkiye; 4 Department of Medical Parasitology, Faculty of Medicine, Kafkas University, Kars, Türkiye

**Keywords:** *Haemaphysalis punctata*, Tick-borne pathogens, Burana virus, *Coxiella burnetii*, *Rickettsia* spp., Anatolia, Türkiye

## Abstract

**Background:**

*Haemaphysalis punctata* is a widespread Palearctic tick species, yet its role in the circulation of tick-borne pathogens in Türkiye remains poorly characterized. No systematic pathogen survey has previously been conducted on host-seeking individuals of this species. This study aimed to provide the first comprehensive molecular investigation of bacterial, protozoan, and viral tick-borne pathogens (TBPs) in questing *H. punctata* populations in Central and Northeastern Anatolia.

**Methods:**

A total of 96 host-seeking adult *H. punctata* were collected from 29 sampling sites in 11 districts across seven provinces. DNA and RNA extracts were screened using a multi-agent polymerase chain reaction (PCR)−-sequencing panel targeting a broad range of TBPs. Positive amplicons were sequenced for species identification, and complete genome sequencing was performed for the detected Burana virus strain. Phylogenetic analyses were conducted using maximum-likelihood and Bayesian approaches.

**Results:**

Microorganisms detected included *Babesia major* (5.21%), *Theileria orientalis* (1.04%), an uncharacterized *Ehrlichia* sp. (1.04%), and spotted fever group rickettsiae (3.13%) comprising *Candidatus* Rickettsia yenbekshikazakhensis and *Rickettsia hoogstraalii*. *Coxiella burnetii* was identified at the highest prevalence (20.83%), representing the first detection of this agent in questing *H. punctata* in Türkiye. Several ticks carried mixed infections. Notably, Burana virus was detected in one specimen—marking the first confirmed occurrence of this orthonairovirus outside Central Asia. Complete S, M, and L segment genomes were recovered, and phylogenetic analyses showed a close—though not identical—relationship to strains from Kyrgyzstan and Xinjiang.

**Conclusions:**

This study provides the first systematic assessment of TBPs in host-seeking *H. punctata* in Türkiye and documents, for the first time in this species, the presence of Burana virus, *Candidatus* R. yenbekshikazakhensis, *T. orientalis*, and *C. burnetii*. The findings highlight *H. punctata* as an underrecognized but epidemiologically relevant tick species in Anatolia and reveal previously undocumented microorganism circulation, with important implications for surveillance of emerging viral and zoonotic threats.

**Graphical Abstract:**

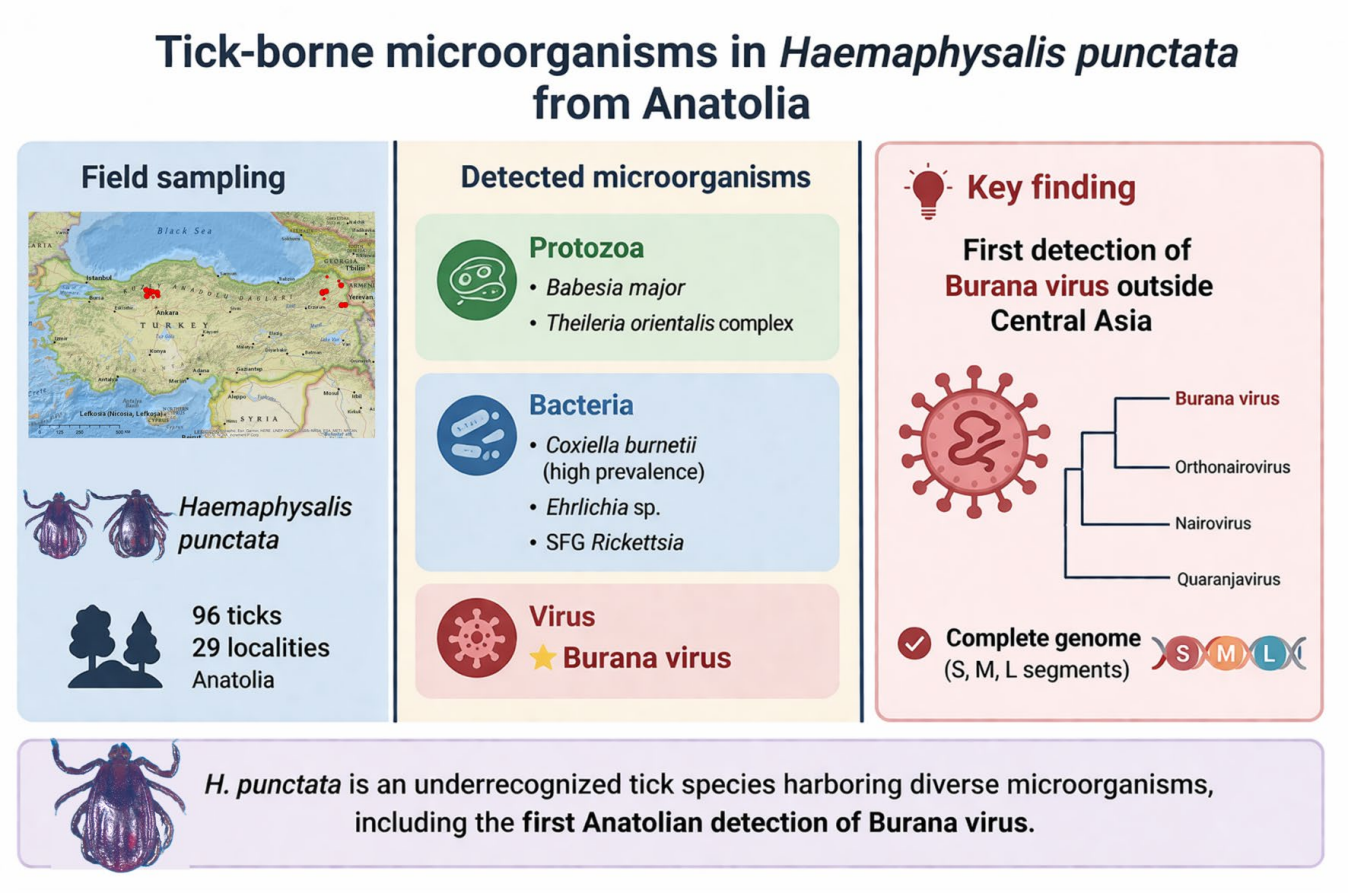

**Supplementary Information:**

The online version contains supplementary material available at 10.1186/s13071-026-07367-4.

## Background

*Haemaphysalis punctata* is well known for its remarkable ability to adapt to a wide spectrum of ecological and climatic conditions, resulting in a broad and continuous distribution throughout the Palearctic region [1, 2]. This exophilic species exhibits a typical three-host life cycle and is capable of completing its development within 1–3 years. Adult ticks display a characteristic bimodal activity pattern, with distinct seasonal peaks occurring during the spring and autumn months [1, 3–5]. Possessing a broad host range, *H. punctata* parasitizes a wide variety of mammals, birds, and even reptiles. Immature stages (larvae and nymphs) commonly feed on small mammals, rabbits, hedgehogs, lizards, and numerous bird species—including migratory taxa—whereas adults predominantly parasitize domestic ruminants such as cattle, sheep, and goats, as well as wild ungulates such as red deer and roe deer [1, 3, 5, 6]. Human infestations have also been documented [7, 8].

Recent observations indicate that *H. punctata* continues to expand its distribution across the Palearctic region, establishing new and previously unrecorded natural foci [9, 10]. The role of migratory birds in facilitating long-distance dispersal, particularly of immature stages, has been highlighted as a major ecological mechanism enabling the species to colonize distant and climatically diverse regions [6]. A striking example of this invasive potential is the unexpected establishment of *H. punctata* populations in Rhode Island, USA—an area far outside its historical distribution—where migratory birds have been suggested as the primary agents of introduction [11].

Historically, *H. punctata* has been associated with several tick-borne pathogens (TBPs) of both veterinary and public health importance, underscoring its relevance as a competent vector species. Reported pathogens include piroplasmids such as *Babesia major* and *B. motasi*; *Francisella tularensis*, the causative agent of tularemia; *Coxiella burnetii*, the agent of Q fever; *Rickettsia sibirica*; *Anaplasma marginale* and *A. centrale*; and viral agents such as Bhanja virus and Tribec virus. Beyond its association with various microorganisms*, H. punctata* has also been implicated in inducing tick paralysis in livestock (particularly sheep, goats, and cattle) and occasionally in humans [1, 5, 6, 12, 13]. The combination of its wide host spectrum, medical and veterinary relevance, and capacity for long-distance dispersal makes *H. punctata* a species of considerable epidemiological significance.

Despite these characteristics, *H. punctata* remains a largely neglected tick species in many regions. Its vector roles within natural foci are insufficiently explored, and consequently, its ecological dynamics and pathogen transmission patterns remain poorly understood. Studies employing comprehensive pathogen screening in host-seeking specimens of this species are particularly scarce. Although *H. punctata* is known to be widely distributed in Anatolia [3, 4, 14], data regarding its vectorial status in this region are extremely limited, and no dedicated study has specifically targeted this species to date. To address these knowledge gaps, the present study aimed to conduct an extensive molecular screening of host-seeking *H. punctata* collected from various sampling sites across Anatolia. By investigating a broad panel of TBPs and characterizing newly detected agents, this study seeks to contribute valuable insights into the vector potential, pathogenic associations, and ecological relevance of *H. punctata* in the region.

## Methods

### Study area, tick collection, and morphological identification

**Fig. 1:**
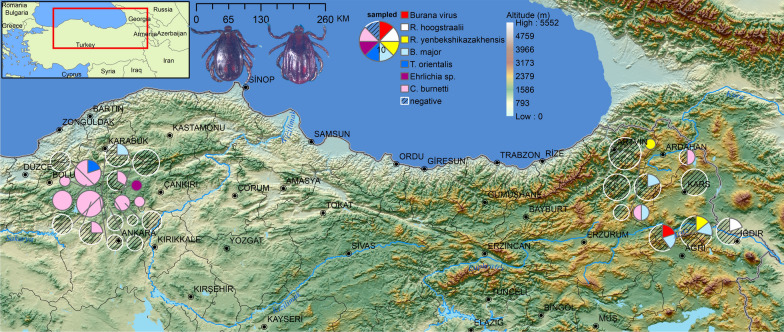
Geographic distribution of host-seeking *Haemaphysalis punctata* collected from Central and Northeastern Anatolia and detection status of tick-borne microorganisms. Each pie chart represents a sampling locality, with color-coded sectors indicating detected viral, bacterial, and protozoan agents based on PCR and sequencing results, while hatched sectors indicate pathogen-negative specimens. Pie size is proportional to the number of ticks analyzed at each locality. Black dots indicate provincial centers and are shown for geographic reference only. Background elevation data (0–5552 m) illustrate major topographic features of the region. Maps were generated using ArcGIS 10.6.1, and the final figure composition was created using Inkscape 1.2

The study was conducted across two geographically and ecologically distinct regions of Türkiye: Central Anatolia and Northeastern Anatolia. These regions differ markedly in terms of altitude, climate, vegetation structure, and dominant land-use patterns. Central Anatolia is characterized by a continental climate with steppe vegetation and moderate elevations, whereas Northeastern Anatolia comprises high-altitude plateaus and mountainous habitats with cooler temperatures and richer forest–meadow mosaics. Host-seeking adult *H. punctata* were sampled within seven provinces (Ankara, Bolu, Erzurum, Kars, Iğdır, Ardahan, and Artvin), covering 11 districts (Kızılcahamam, Çamlıdere, Çubuk, Gerede, Şenkaya, Kağızman, Arpaçay, Sarıkamış, Tuzluca, Çıldır, and Şavşat). A total of 29 sampling sites representing diverse ecological conditions were surveyed (Fig. 1; Table 1).

**Table 1:** Geographical distribution of sampled host-seeking *Haemaphysalis punctata* and results of molecular screening for tick-borne pathogens across Anatolia

Province	District	No. of locations	No. of tested ticks	*Babesia* spp.	*Theileria* spp.	*Hepatozoon* spp.	*Anaplasma* spp.	*Borrelia* spp.	*Rickettsia* spp.	*Ehrlichia* spp.	*Coxiella* spp.	*Francisella* spp.	CCHFV	Nairo-generic
Ankara	Kızılcahamam	7	18 (10♀, 8♂)	1	−	−	−	−	−	1	1	−	−	−
	Çamlıdere	5	17 (7♀, 10♂)	−	−	−	−	−	−	−	6	−	−	−
	Çubuk	3	6 (6♂)	−	−	−	−	−	−	−	3	−	−	−
Bolu	Gerede	3	9 (7♀, 2♂)	−	1^c^	−	−	−	−	−	8^e^	−	−	−
Erzurum	Şenkaya	4	19 (15♀, 4♂)	1	−	−	−	−	−	−	−	−	−	−
Kars	Kağızman	2	12 (8♀, 4♂)	2^a^	−	−	−	−	1^d^	−	−	−	−	1^ g^
	Arpaçay	1	5 (4♀, 1♂)	−	−	−	−	−	−	−	−	−	−	−
	Sarıkamış	1	2 (2♀)	1^b^	−	−	−	−	−	−	1^f^	−	−	−
Iğdır	Tuzluca	1	5 (2♀, 3♂)	−	−	−	−	−	1	−	−	−	−	−
Ardahan	Çıldır	1	2 (2♀)	−	−	−	−	−	−	−	1	−	−	−
Artvin	Şavşat	1	1 (1♂)	−	−	−	−	−	1	−	−	−	−	−
Total	11	29	96 (57♀, 39♂)	5 (5.21%)	1 (1.04%)	0	0	0	3 (3.12%)	1 (1.04%)	20 (20.83%)	0	0	1 (1.04%)

♂: Male, ♀: Female

^a^One of *Babesia*-positive ticks was found as mixed with rickettsiae and the other one with Nairo virus

^b^One *Babesia*-positive tick was found as mixed with *Coxiella* spp.

^c^One *Theileria*-positive tick was found as mixed with *Coxiella* spp.

^d^One *Rickettsia*-positive tick was found as mixed with *Babesia* spp.

^e^One of *Coxiella*-positive ticks was found as mixed with *Theileria* spp.

^f^One *Coxiella*-positive tick was found as mixed with *Babesia* spp.

^g^One Nairo virus-positive tick was found as mixed with *Babesia* spp.

Sampling was carried out during the spring and autumn activity periods of *H. punctata* in 2022, and during the spring season of 2023. Ticks were collected using the flagging method, whereby a 1.5 × 1 m^3^ white cotton cloth was swept over vegetation to capture actively questing adults. When observed directly in the field, additional specimens were collected by hand. Each tick was placed into aerated, location-labeled collection vials, and associated geographical information was recorded. Specimens collected in Central Anatolia were transported alive to the Ticks and Tick-borne Diseases Research Laboratory (TTBDRL) at Ankara University Faculty of Veterinary Medicine on the same day. Ticks collected from Northeastern Anatolia were initially delivered to the Department of Parasitology at Kafkas University under appropriate conditions and subsequently transferred alive to TTBDRL within 1 week.

All specimens were morphologically identified to species level under a stereomicroscope (Stemi 2000-C, Zeiss, Germany; equipped with an AxioCam digital camera and ZEN software), using standard taxonomic keys [15]. Following identification, each tick was washed briefly in 70% ethanol, rinsed thoroughly with sterile DNase/RNase-free water, and air-dried on sterile filter paper. The ticks were then individually placed in sterile tubes and stored at –80 °C until molecular analyses were performed.

### Nucleic acid extraction, cDNA synthesis, and PCR

Each adult *H. punctata* specimen was processed individually for nucleic acid extraction. Ticks were mechanically homogenized in bead-containing tubes using a SpeedMill PLUS cooling homogenizer (Analytik Jena, Jena, Germany) to ensure efficient disruption of tissues. Genomic DNA and total RNA were subsequently isolated from the homogenates using the InnuPREP Tick DNA/RNA Kit (IST Innuscreen GmbH, Berlin, Germany) following the manufacturer’s protocol. DNA extracts were stored at –20 °C, whereas RNA samples were preserved at –80 °C until further analyses. Complementary DNA (cDNA) was synthesized from total RNA using the Wonder RT cDNA Synthesis Kit (Euroclone, Pero, Italy) and kept at –20 °C prior to PCR applications.

To verify sample integrity and detect potential PCR inhibitors, all DNA extracts were first screened using a tick-specific internal control targeting the mitochondrial 16S ribosomal RNA (rRNA) gene [16]. Only specimens yielding successful amplification were included in downstream microorganism screening. Both DNA and cDNA templates were subsequently subjected to an extensive panel of conventional and nested PCR assays designed to detect a broad range of bacterial, protozoan, and viral TBPs [17–35]. Primer sets targeting the selected microorganisms and detailed PCR cycling conditions are provided in Supplementary Table 1.

Each PCR run included sterile DNase/RNase-free water as a negative control and pathogen-specific positive controls to ensure assay performance. Positive control material comprised DNA or cDNA from reference organisms including *A. phagocytophilum*, *A. ovis*, *B. divergens*, *Borrelia afzelii*, *C. burnetii*, *Ehrlichia* sp., *F. tularensis*, *Hepatozoon felis*, *R. montanensis*, *Theileria annulata*, and Crimean–Congo hemorrhagic fever virus (CCHFV). To enhance sensitivity and minimize false-negative outcomes in low-copy-number targets, pilot PCR runs using serial dilutions of positive controls (1:1–1:100 for conventional PCRs and 1:1–1:1000 for nested PCRs) were conducted to optimize amplification conditions.

### Sequencing and phylogenetic analyses

All PCR amplicons obtained from the microorganism screening assays were purified using the ExoSAP-IT™ PCR Product Cleanup Kit (Thermo Scientific, Santa Clara, CA, USA) and subjected to Sanger sequencing. Bidirectional sequencing reactions were performed using the BigDye™ Terminator v3.1 Cycle Sequencing Kit (Applied Biosystems, Foster City, CA, USA) on an Applied Biosystems™ 3500 Genetic Analyzer. Chromatograms were visually inspected, edited, and assembled into consensus sequences using AliView version 1.26 [36]. The resulting sequences were compared with reference sequences in GenBank through BLAST analyses to assign taxonomic identity. For each microorganism group, haplotype datasets were generated and diversity indices were calculated using DnaSP version 6.12.03 [37].

One specimen that yielded a positive result with the nairovirus-generic PCR (partial L-segment) was identified as Burana virus (*Orthonairovirus buranaense*) on the basis of BLAST similarity. To obtain the full viral genome, total RNA extracted from this tick was processed for next-generation sequencing (NGS). RNA quality and quantity were assessed spectrophotometrically (NanoDrop ND-1000, Thermo Scientific) and fluorometrically (Qubit 3, Thermo Scientific), and structural integrity was evaluated using the 4200 TapeStation (Agilent, USA). Library preparation was carried out using the Illumina Total RNA Prep Kit (Illumina, USA), which includes rRNA depletion, random fragmentation, purification, first- and second-strand cDNA synthesis, adaptor/index ligation, and final library cleanup. Sequencing was performed on an Illumina NovaSeq 6000 platform, generating approximately 10 million paired-end reads (150 bp) for the specimen. Library quantification, dilution, and loading were conducted according to the manufacturer’s guidelines.

Quality assessment of raw NGS reads was performed using FASTQC, which provided metrics on read quality, GC content, *k*-mer distribution, and potential adaptor contamination. Low-quality bases and adaptor-derived sequences were removed using Trimmomatic version 0.39 [38] to obtain high-quality reads for downstream processing. Filtered reads were then assembled de novo using Shovill version 1.1.0, integrating SPAdes, SKESA, and Velvet assemblers [39]. Assembly metrics—including total contig number, N50, L50, mean contig length, total assembly size, and GC content—were evaluated with QUAST version 5.3.0 [40]. SPAdes provided the highest-quality assembly, and the resulting contigs were screened against reference Burana virus segments (NC_043439.1, NC_043438.1, and NC_043437.1) using BLASTn. Three contigs showed high-confidence matches to the L, M, and S genome segments of Burana virus.

For phylogenetic reconstruction of bacterial and protozoan microorganisms, multiple loci were analyzed. Partial 18S rRNA sequences were used for *Babesia* and *Theileria* spp.; *com1* and IS1111-Tnp for *Coxiella*; *ompA*, *ompB*, and *gltA* for spotted fever group (SFG) rickettsiae; and *groEL* for *Ehrlichia*. Sequence reliability and alignment quality were assessed using GUIDANCE2 [41], and low-confidence residues or sequences were removed prior to downstream analyses. Noncoding genes (e.g., 18S rRNA) were aligned using the Q-INS-i algorithm implemented in MAFFT version 7.475 [42], while translated alignments for protein-coding genes (*com1*, *groEL*, *ompA*, *ompB*, and *gltA*) were generated using MUSCLE [43] embedded within AliView. Alignment quality and evolutionary divergence were evaluated using *p*-distance estimates in MEGA version 11.0.13 [44]. Bayesian inference was used for all bacterial and protozoan phylogenies. The best-fit nucleotide substitution models were selected under the Bayesian information criterion (BIC) using jModelTest version 2.1.10 [45]. Analyses were performed in BEAST2 (BEAUti version 2.7.6, BEAST version 2.7.6, and TreeAnnotator version 2.7.4) [46] using Markov chain Monte Carlo (MCMC) runs of 100 million generations. Convergence and effective sample sizes (ESS > 200) were verified in Tracer version 1.7.2 [47]. Maximum clade credibility (MCC) trees were generated after discarding the first 20% of sampled trees as burn-in and visualized in FigTree version 1.4.4. For Burana virus, amino acid-based phylogenetic analyses were performed separately for each genome segment (L, M, and S). Maximum-likelihood (ML) phylogenies were constructed using IQ-TREE 3 [48], with model selection performed automatically via ModelFinder [49] and branch support estimated using 1000 ultrafast bootstrap replicates. The resulting trees were compared with representative nairovirus sequences to determine the evolutionary position of the newly generated Burana virus genome.

### Mapping and database registration

The geographical distribution of the detected microorganisms was visualized using ArcGIS 10.6.1 (Esri, 2018). In addition, all haplotype sequences generated in this study were deposited in GenBank under the corresponding accession numbers: *B. motasi* (PX600294–PX600298), *Theileria orientalis* (PX600299), *Candidatus* Rickettsia yenbekshikazakhensis (PX620503–PX620504), *Rickettsia hoogstraalii* (PX620505), *Ehrlichia* sp. (PX620506), *C. burnetii* (PX620507–PX620510), and Burana virus (PX620511–PX620513) (Table 2).

**Table 2:** Tick-borne pathogens detected in host-seeking *Haemaphysalis punctata* specimens from Central and Northeastern Anatolia, and their nucleotide sequence identities with closely related GenBank entries

Detected pathogens		No. of positive tick specimens (sex)	District/province (no. of positive ticks)	Sequenced gene	Haplotype	Nucleotide identity percentage (%)	GenBank accession no.
*Babesia* spp.	*B. major*	5 (2♀, 3♂)	Kağızman/Kars (1) Şenkaya/Erzurum (1) Sarıkamış/Kars (1) Kağızman/Kars (1) Kızılcahamam/Ankara (1)	18S rRNA	Bma-HpuHp1 Bma-HpuHp2 Bma-HpuHp3 Bma-HpuHp4 Bma-HpuHp5	99.77^a,b^ 99.53^a,c^ 99.77^d,e^ 99.77^f^ 99.30^a,b,c^	PX600297 PX600295 PX600298 PX600294 PX600296
*Theileria* spp.	*T. orientalis*	1 (♀)	Gerede/Bolu (1)	18S rRNA	Tor-HpuHp1	100^ g^	PX600299
*Rickettsia* spp.	*R. hoogstraalii* *C.* R. yenbekshikazakhensis	1 (♀) 2 (♂)	Tuzluca/Iğdır (1) Kağızman/Kars (1) Şavşat/Artvin (1)	*gltA* *ompA* + *ompB* *ompA* + *ompB*	Rho-HpuHp1 Rye-HpuHp1 Rye-HpuHp1	100^ h^ 98.51^ı,j^/100^ k^ 98.51^ı,j^/100^ k^	PX620505 PX620503/PX620504 PX620503/PX620504
*Ehrlichia* spp.	*Ehrlichia* sp.	1 (♂)	Kızılcahamam/Ankara (1)	*groEL*	Ehg-HpuHp1	96.46^ l^	PX620506
*Coxiella* spp.	*C. burnetii*	20 (12♀, 8♂)	Çamlıdere/Ankara (6) Çubuk/Ankara (2) Çubuk/Ankara (1) Kızılcahamam/Ankara (1) Çıldır/Ardahan (1) Gerede/Bolu (8) Sarıkamış/Kars (1)	ISI1111-Tnp + com1 ISI1111-Tnp + com1 ISI1111-Tnp + com1 ISI1111-Tnp + com1 ISI1111-Tnp + com1 ISI1111-Tnp + com1	Cbi-HpuHp2/Cbc-HpuHp2 Cbi-HpuHp2/Cbc-HpuHp2 Cbi-HpuHp2/Cbc-HpuHp1 Cbi-HpuHp2/Cbc-HpuHp2 Cbi-HpuHp1/Cbc-HpuHp2 Cbi-HpuHp2/Cbc-HpuHp2 Cbi-HpuHp2/Cbc-HpuHp2	100^ m^/99.78^n,o^ 100^ m^/99.78^n,o^ 100^ m^/99.79^p^ 100^ m^/99.78^n,o^ 99.81^ m^/99.78^n,o^ 100^ m^/99.78^n,o^ 100^ m^/99.78^n,o^	PX620510/PX620508 PX620510/PX620508 PX620510/PX620507 PX620510/PX620508 PX620509/PX620508 PX620510/PX620508 PX620510/PX620508
Nairoviridae	Burana virus (*Orthonairovirus buranaense*)	1 (♂)	Kağızman/Kars (1)	S-segment complete M-segment complete L-segment complete	Bur-Hpu/Lc-1934/2022/TR	98,51^q^ 97,77^r^ 98,71^ s^	PX620511 PX620512 PX620513

^a^*Babesia major* clone Yili20, accession no. MF120940

^b^*Babesia major* isolate RT6-Cildir, accession no. OR211414

^c^*Babesia major* isolate Yili, accession no. AY603399

^d^*Babesia major* isolate Bma-Sokuluk-Cattle44, accession no. LC768119

^e^*Babesia major* isolate RT5-Selim, accession no. OR211413

^f^*Babesia major* isolate BB-86/Ank-Ha.punct, accession no. KF791206

^g^*Theileria orientalis* isolate Th_orient_BiH3, accession no. ON148462

^h^*Rickettsia hoogstraaliii* TR-Rg344, accession no. MK929316

^ı^*Rickettsia aeschlimannii* isolate Ningxia-Ha-3, accession no. PQ122916

^j^*Rickettsia aeschlimannii* isolate Ro-775, accession no. MF379308

^k^*Candidatus* Rickettsia yenbekshikazakhensis isolate Tekeli_093, accession no. MG973879

^l^*Ehrlichia* sp*.* clone Kh-Hj27, accession no. FJ966349

^m^*Coxiella burnetii* strain EVC282, accession no. KT391019

^n^*Coxiella burnetii* strain Nine Mile Q, accession no. CP177352

^o^*Coxiella burnetii* strain CbTR-DmHp1, accession no. PP465022

^p^*Coxiella burnetii* strain Ko Q229, accession no. AB004702

^q^Burana virus strain 760, accession no. NC_043437

^r^Burana virus strain 760, accession no. NC_043438

^s^Burana virus strain 760, accession no. NC_043439

## Results

### Tick collection and screening overview

A total of 96 host-seeking adult *H. punctata* (57♀, 39♂), collected from 29 sampling sites across 11 districts in seven provinces of Central and Northeastern Anatolia, were screened for TBPs. PCR analyses revealed positive amplifications for several microorganism groups. *Babesia*-specific PCR assays were positive in five samples (5.21%), whereas *Theileria*-specific PCR yielded one positive sample (1.04%). Among bacterial agents, SFG *Rickettsia* PCRs were positive in three individuals (3.13%), and *Ehrlichia*-targeted PCR assays in one individual (1.04%). *Coxiella*-specific PCRs produced positive results in 20 ticks, representing the highest prevalence detected in this study (20.83%). In addition, one tick (1.04%) was positive in the nairovirus-generic PCR assay.

Mixed infections were also detected: among the five *Babesia* PCR-positive ticks, one was concurrently positive in the *Rickettsia* PCR, one in the *Coxiella* PCR, and one in the nairovirus-generic PCR. The single *Theileria* PCR–positive tick was also positive for *Coxiella*. No PCR amplification indicative of *Anaplasma*, *Hepatozoon*, *Francisella*, CCHFV, or *Borrelia burgdorferi* sensu lato was obtained.

### Protozoan microorganisms

#### *Babesia* spp.

Sequencing of all five *Babesia*-positive PCR amplicons revealed that each belonged to *B. major*. Four of the *B. major*-positive ticks (two males and two females) originated from four distinct sampling sites in Northeastern Anatolia (two from Kağızman, one from Sarıkamış, and one from Şenkaya district), while the remaining sample (male) was collected from a single site in Central Anatolia (Kızılcahamam district) (Table 2).

Haplotype analysis based on partial 18S rRNA sequences demonstrated that all five *B. major*-positive ticks represented distinct haplotypes (Bma-HpuHp1 to Bma-HpuHp5), indicating high genetic diversity among the *B. major* isolates. BLAST comparisons showed that none of these haplotypes were identical to any sequence currently available in GenBank, confirming that all were unique. Bma-HpuHp1 exhibited its highest similarity (99.72%; 428/429 bp) to sequences obtained from *H. punctata* in China (MF120940) and from cattle in Ardahan, Türkiye (OR211414). Bma-HpuHp2 showed 99.53% similarity (426/428 bp) to two *H. punctata*-derived sequences from China (MF120940 and AY603399). Bma-HpuHp3 exhibited 99.77% similarity (429/430 bp) to *B. major* sequences from cattle in Kyrgyzstan (LC768119) and Kars, Türkiye (OR211413). Bma-HpuHp4 showed 99.77% similarity (428/429 bp) to a sequence previously detected in *H. punctata* from Ankara, Türkiye (KF791206). Bma-HpuHp5 displayed 99.30% similarity (425/428 bp) to sequences from Ardahan, Türkiye (OR211414) and two Chinese isolates (MF120940 and AY603399). Mixed infections were also observed among *B. major*-positive ticks. One of the five samples was concurrently infected with *Candidatus* R. yenbekshikazakhensis, another with *C. burnetii*, and a third with Burana virus (Table 2).

Bayesian phylogenetic analysis of partial 18S rRNA sequences revealed that the *B. major* clade was divided into two subclades with maximal posterior support (posterior = 1). Two haplotypes identified in this study (Bma-HpuHp1 and Bma-HpuHp4) grouped within one subclade, while the remaining three (Bma-HpuHp2, Bma-HpuHp3, and Bma-HpuHp5) clustered within the second subclade (Supplementary Fig. S1).

#### *Theileria* spp.

Sequencing of the single *Theileria*-positive sample identified the agent belonging to the *Theileria orientalis* complex (hereafter *T. orientalis*). This sample (female) originated from a sampling site in the Gerede District of Central Anatolia. BLAST analysis showed that the *T. orientalis* haplotype (Tor-HpuHp1) was identical to several GenBank sequences, including records from cattle in Bosnia and Herzegovina (ON148462) and from a water buffalo in Iğdır, Türkiye (PX446659). The *T. orientalis*-positive tick exhibited a mixed infection with *C. burnetii* (Table 2).

Bayesian inference analysis based on partial 18S rRNA sequences demonstrated that the *T. orientalis* haplotype generated in this study clustered robustly within the main *T. orientalis* clade. The sequence grouped together with other *T. orientalis* isolates reported from Türkiye (Supplementary Fig. S1).

### Bacterial microorganisms

#### *Ehrlichia* spp.

PCR screening targeting the 16S rRNA gene yielded a single *Ehrlichia*-positive sample. This tick (male) originated from a sampling site in the Kızılcahamam district of Central Anatolia. Given the highly conserved nature of the 16S rRNA gene within the genus, a more variable locus—the partial *groEL* gene—was amplified and sequenced to enable species-level characterization. Sequence analysis of the *groEL* amplicon revealed that the detected microorganism represented an undescribed *Ehrlichia* species. BLAST comparisons indicated that the obtained *groEL* haplotype had no identical matches in GenBank. The closest sequence (96.46% similarity; 573/594 bp) corresponded to an unclassified *Ehrlichia* genotype previously detected in *Haemaphysalis japonica* from Russia (FJ966349) (Table 2).

Bayesian phylogenetic inference based on the *groEL* gene demonstrated that the haplotype identified in this study clustered within the main *Ehrlichia* clade and formed a separate, well-supported lineage (posterior = 0.93). This lineage was monophyletic with two *Ehrlichia* genotypes previously reported from *H. japonica* and *Haemaphysalis concinna* in Russia (FJ966349 and JX092091). In addition, the sequence showed close evolutionary affinity to an *Ehrlichia* genotype detected in *Haemaphysalis muhsami* from the Democratic Republic of the Congo (Supplementary Fig. S2).

#### *Coxiella* spp.

PCR analysis targeting the IS1111-Tnp insertion sequence revealed *Coxiella*-positivity in 20 *H. punctata* individuals. In addition, amplification of the more informative and protein-coding *com1* gene yielded positive amplicons in all 20 samples. Of these *Coxiella*-positive ticks, 18 (10 females and eight males) originated from nine distinct sampling sites in Central Anatolia (including Gerede [*n* = 8], Çamlıdere [*n* = 6], Çubuk [*n* = 3], and Kızılcahamam [*n* = 1] districts), while two (females) originated from Northeastern Anatolia (Çıldır and Sarıkamış districts) (Table 2).

Sequence analysis of both *com1* and IS1111-Tnp amplicons demonstrated that all 20 samples belonged to *C. burnetii*. Haplotype analysis revealed the presence of two haplotypes for each gene. For the *com1* locus, one haplotype (Cbc-HpuHp1) was represented solely by the Çubuk-derived sample, whereas the remaining 19 samples clustered into a second haplotype (Cbc-HpuHp2). The two haplotypes differed by a single nucleotide substitution. BLAST analysis indicated that neither haplotype had an identical match in GenBank. Cbc-HpuHp1 showed its highest similarity (99.79%, 475/476 bp) to an isolate of human from Canada (AB004702), whereas Cbc-HpuHp2 exhibited 99.78% similarity (455/456 bp) to several records, including *C. burnetii* from *Dermacentor andersoni* in the USA (CP177352) and a sequence previously reported from host-seeking *Dermacentor marginatus* in Türkiye (PP465022). Similarly, IS1111-Tnp sequences formed two haplotypes: the Çıldır-derived sample represented a distinct haplotype (Cbi-HpuHp1), while all remaining specimens shared a second haplotype (Cbi-HpuHp2). As in the *com1* dataset, the two haplotypes differed by a single nucleotide. BLAST analysis revealed that Cbi-HpuHp1 had no identical sequence in GenBank and showed its highest similarity (99.81%, 524/525 bp) to a *C. burnetii* isolate obtained from a goat in France (KT39019), whereas Cbi-HpuHp2 was identical to this French isolate. Mixed infections were also detected among *C. burnetii*-positive ticks. One *C. burnetii*-positive sample from Gerede District was concurrently infected with *B. major*, and another from Sarıkamış District with *T. orientalis* (Table 2).

Bayesian phylogenetic analyses of both markers showed that the haplotypes generated in this study clustered firmly within the principal *C. burnetii* clade, alongside a diverse array of isolates from multiple countries and host species. In the *com1* phylogeny, the main *C. burnetii* lineage was subdivided into two strongly supported subclades (posterior = 1), with both haplotypes (Cbc-HpuHp1 and Cbc-HpuHp2) positioned together within one of these subclades (Supplementary Fig. S3). In contrast, the IS1111-Tnp phylogeny did not exhibit well-supported subclade structure; however, both haplotypes again clustered closely together within the broader *C. burnetii* clade (Supplementary Fig. S4).

#### *Rickettsia* spp.

PCR screening targeting the *gltA* gene yielded *Rickettsia*-positive amplification in three *H. punctata* individuals. All three positive ticks (one female and two males) originated from Northeastern Anatolia, each from a distinct sampling site located in Tuzluca, Kağızman, and Şavşat districts. To achieve more refined taxonomic resolution, *ompA* PCR—which targets a more variable locus—was subsequently performed. Successful *ompA* amplification was obtained from the Kağızman- and Şavşat-derived samples, whereas the Tuzluca specimen did not yield an *ompA* product. Thus, the *gltA* amplicon from the Tuzluca sample and the *ompA* amplicons from the other two samples were sequenced (Table 2).

BLAST analysis of the *gltA* sequence from the Tuzluca sample (female) demonstrated that it was identical to multiple *R. hoogstraalii* sequences available in GenBank, including several records previously reported from *Haemaphysalis parva* in Türkiye (e.g., MK9293916, MF379276, and JQ691712). In contrast, *ompA* sequence analysis of the Kağızman and Şavşat samples (males) indicated that both belonged to the same haplotype, although this haplotype did not match any sequence currently deposited in GenBank. The highest similarity (98.51%; 594/603 bp) was observed with several *Rickettsia aeschlimannii* sequences, but this level of divergence and the lack of complete identity indicated that the agent could not be reliably assigned to *R. aeschlimannii* on the basis of *ompA* alone.

Given the high variability of *ompA*, the *ompB* gene was subsequently amplified and sequenced to further resolve the identity of these two *Rickettsia* specimens. *ompB* sequence analysis revealed that both ticks shared a single haplotype, which was found to be identical to multiple *Candidatus* R. yenbekshikazakhensis sequences previously obtained from *H. punctata* specimens in Kazakhstan (MG973879, MG973893, MG973896, and MG973907). These findings confirm that two of the *Rickettsia*-positive *H. punctata* specimens detected in this study carried *Candidatus* R. yenbekshikazakhensis. One of these (originating from Kağızman) exhibited mixed infection with *B. major* (Table 2).

Bayesian phylogenetic analysis of *ompB* sequences demonstrated that the haplotype identified in this study clustered within the well-defined *Candidatus* R. yenbekshikazakhensis clade, together with isolates from Kazakhstan and Türkiye (Supplementary Fig. S5). In contrast, Bayesian inference based on the *ompA* gene showed the Turkish *Candidatus* R. yenbekshikazakhensis haplotype forming its own distinct lineage in the absence of previously deposited reference sequences for this gene. This lineage was strongly supported (posterior = 0.99) and formed a monophyletic grouping with *R. aeschlimannii*, reflecting the close—but clearly distinct—relationship between these taxa at the *ompA* locus (Supplementary Fig. S6).

### Viral microorganisms

#### Burana virus

PCR screening targeting the partial L segment of Nairoviridae yielded a single positive amplification in a *H. punctata* specimen (male) collected from a sampling site in Kağızman District of Northeastern Anatolia. Sequence analysis of this partial L-segment amplicon confirmed that the detected virus belonged to Burana virus. BLAST results showed that the partial sequence shared 97.37–98.03% identity (445–448/457 bp) with Burana virus sequences previously reported from *H. punctata* in Kyrgyzstan and Xinjiang, China. The closest non-Burana match was Shanxi tick virus 2 (OR114980), with markedly lower identity (78.65%, 361/459 bp; four gaps). Additionally, this Burana virus-positive tick was found to be coinfected with *B. major*.

Whole-genome sequencing of the positive specimen successfully recovered complete S, M, and L segments. The complete S segment was 1755 nt in length and contained a single major open reading frame (ORF) encoding the nucleoprotein (N). This principal ORF extended from nucleotide positions 88–90 to 1567–1569, producing a 1482-nt coding region corresponding to a predicted 494-aa protein (~ 84% of the segment). The 5′ and 3′ untranslated regions (UTRs) measured 87 nt and 186 nt, respectively. A secondary short ORF in an alternative reading frame was also identified near the 3′ terminus (positions 1505–1507 to 1682–1684), encoding a putative 60-aa peptide. BLAST analysis revealed that the S-segment showed its highest similarity (98.51%, 1457/1479 bp) to the Burana virus type strain isolated from *H. punctata* in Kyrgyzstan (NC_043437).

The complete M segment was 4483 nt long and contained a single dominant ORF spanning positions 312–4347, encoding a 4032-nt glycoprotein precursor (1344 aa). This genomic organization was consistent with orthonairovirus M-segments, including a signal peptide at the N-terminus and conserved motifs associated with Gn/Gc maturation. The 5′ UTR measured 311 nt, and the 3′ UTR measured 136 nt. BLAST comparison showed 97.77% identity (3942/4032 bp) to the Burana virus M-segment reference from Kyrgyzstan (NC_043438).

The L segment was 12,059 nt in length and encoded a single large ORF consistent with the RNA-dependent RNA polymerase (RdRp). This ORF extended from nucleotide positions 57–59 to 11,974–11,976, producing an 11,920-nt coding region corresponding to a predicted 3972-aa polymerase. Terminal untranslated regions were short (57-nt 5′ UTR and 83-nt 3′ UTR), matching the compact architecture typical for orthonairovirus L-segments. BLAST analysis demonstrated that the L-segment shared 98.71% identity (11,762/11,916 bp) with the Kyrgyzstan Burana virus reference sequence (NC_043439).

**Fig. 2:**
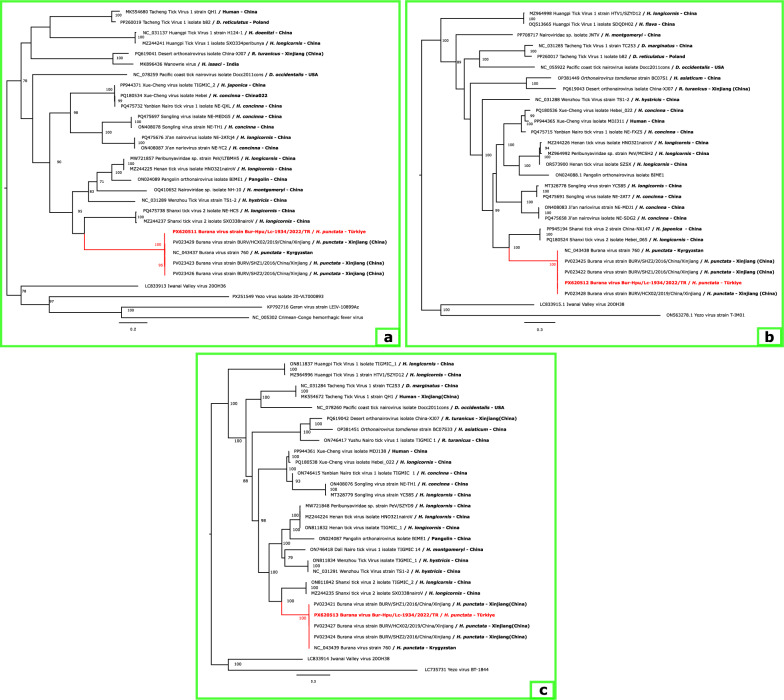
Maximum-likelihood phylogenetic placement of the Burana virus strain detected in this study based on complete amino-acid sequences of the three genomic segments: **a** S-segment (nucleoprotein), inferred under the LG + I + G4 substitution model; **b** M-segment (glycoprotein precursor), inferred under the Q.insect + R4 model; and **c** L-segment (RNA-dependent RNA polymerase), inferred under the Q.insect + F + I + R4 model. The sequence obtained from a host-seeking *Haemaphysalis punctata* in Türkiye (Bur-Hpu/Lc-1934/2022/TR) is highlighted in red and consistently clusters within the Burana virus clade. GenBank accession numbers are provided before virus names, and collection source information with country of origin is shown after isolate names. Node support values ≥ 70% are indicated. Scale bars represent substitutions per site

Maximum-likelihood phylogenies generated for amino acid datasets of all three segments (S, M, and L) consistently placed the newly sequenced isolate within the Burana virus clade, clustering together with sequences from Kyrgyzstan and Xinjiang, China. Across all segment trees, Burana virus formed a well-supported monophyletic group that showed a sister-clade relationship to Shanxi tick virus 2, supported by high bootstrap values (Fig. 2).

## Discussion

*Haemaphysalis punctata* is a widely distributed Palearctic tick occupying diverse habitats from lowland pastures to mountainous ecosystems [1–3]. Owing to its frequent infestation of domestic animals and occasional feeding on humans, it is regarded as a tick species of medical and veterinary relevance [5, 9–11]. Nevertheless, despite its broad geographic range across Europe, the Caucasus, the Middle East, Central Asia, and North Africa [2, 50], *H. punctata* has remained comparatively understudied in TBP research. Most published data derive from geographically limited or host-derived collections and largely represent incidental detections rather than structured surveys [51–65]. In particular, investigations targeting host-seeking *H. punctata* are rare [9, 66–68], resulting in its underrepresentation compared with other sympatric ixodid ticks such as *Hyalomma*, *Dermacentor*, and *Ixodes*. This knowledge gap persists despite the species’ capacity for long-distance dispersal via migratory birds and terrestrial host movements, highlighting the need for focused epidemiological studies.

In Türkiye, *H. punctata* is known to infest a wide range of hosts—including cattle, sheep, goats, small mammals, birds, and humans [3, 4, 7, 14, 69–74]—yet its vector competence and pathogen repertoire have never been comprehensively evaluated. In Türkiye, pathogen detections in *H. punctata* have so far been limited to isolated findings from host-derived ticks, including *B. major* in a specimen removed from a human and an undescribed *Ehrlichia* genotype in a tick collected from sheep [54, 63]. Importantly, no systematic screening has previously been conducted on host-seeking *H. punctata*. Beyond these sparse observations, data on TBPs carried by this species in the region are essentially absent. Given this substantial knowledge gap, the present study was designed to provide a focused and systematic assessment of TBPs in host-seeking *H. punctata* across ecologically diverse sampling sites of Central and Northeastern Anatolia.

### First detection and full-genome characterization of Burana virus outside Central Asia

Burana virus is a Tamdy-group orthonairovirus that was originally described from an isolate (LEIV-Krg760) obtained in 1971 from *H. punctata* collected from cattle in the Tokmak/Burana region of Kyrgyzstan [75, 76]. It is currently assigned the species *O. buranaense*. Despite its formal taxonomic recognition, knowledge of the ecology and geographic distribution of Burana virus has remained almost entirely restricted to this historical Central Asian focus [76–78]. More recently, metagenomic surveys revealed Burana virus-related sequences in *H. punctata* specimens from Xinjiang, China, suggesting that the virus may be more widely distributed than previously assumed [79]. However, prior to the present work, no occurrence outside Central Asia had been confirmed.

This study provides the first confirmed detection and full-genome characterization of Burana virus in Anatolian host-seeking *H. punctata*, thereby substantially expanding its known geographic range westwards into the Palearctic region. BLAST analyses revealed high—but not complete—nucleotide identity (~ 97.7–98.7%) to the reference BURV strains from Kyrgyzstan and Xinjiang, indicating that the Anatolian sequence belongs to the same virus species yet likely represents a regionally adapted and independently evolving lineage. Maximum-likelihood phylogenies based on the amino-acid sequences of all three genomic segments consistently placed the detected strain within the Burana virus monophyletic cluster, showing strong support for a close evolutionary relationship with the Chinese and Kyrgyz isolates. In each tree, this cluster formed a robust sister relationship to Shanxi tick virus 2, further highlighting the distinct diversification trajectory of Tamdy-group orthonairoviruses.

Epidemiologically, the detection in a host-seeking adult tick strongly indicates that Burana virus is circulating independently of a recent bloodmeal, suggesting stable transstadial maintenance within local *H. punctata* populations. In the two previous records of Burana virus, the virus was detected in *H. punctata* originating from a cattle host in Kyrgyzstan and from uncharacterized sampling sources in Xinjiang, China [76, 79]. Thus, the present study constitutes the first clear evidence of Burana virus infection in a genuinely questing *H. punctata*, demonstrating that the virus is maintained in the tick body beyond detachment from a vertebrate host. This provides an important epidemiological indication that *H. punctata* may serve not only as an incidental carrier but as a biologically involved vector in natural Burana virus transmission cycles. The fact that the same tick also harbored *B. major* underscores the ability of *H. punctata* to simultaneously carry pathogens with very different biological and ecological traits, raising the possibility of overlapping or interacting transmission cycles among local wildlife and livestock communities.

To date, no confirmed clinical disease has been attributed to Burana virus in either humans or animals. However, Tamdy-group orthonairoviruses have recently gained attention as emerging arboviruses, with human febrile illness reported following exposure to related Tamdy virus (*Orthonairovirus tomdiense*) [80] and experimental pathogenicity demonstrated for Shanxi tick virus 2 in neonatal mouse models [81]. Given the strong phylogenetic affinity of Burana virus to Shanxi tick virus 2, continued surveillance is therefore warranted. Moreover, the geographic pattern of detections to date—Kyrgyzstan, Xinjiang and now Anatolia—closely follows the Palearctic distribution of *H. punctata*, strongly suggesting that the virus’ epidemiology is intrinsically linked to the ecology of this tick species and may extend wherever *H. punctata* persists. The discovery of a complete infectious genome outside Central Asia demonstrates that Burana virus is not an isolated regional virus, but part of a broader, likely underrecognized nairovirus system spanning *H. punctata* habitats from Central Asia into Anatolia. These findings highlight the need to incorporate Burana virus into future tick-borne virus surveillance and differential diagnostic strategies in Türkiye.

Overall, our findings (i) expand the recognized biogeographical range of Burana virus, (ii) demonstrate its active circulation in a previously unsurveyed region, and (iii) identify *H. punctata* as a primary target for future virological investigations. To fully assess the zoonotic potential and epidemiological relevance of this orthonairovirus, virus isolation, vector-competence experimentation, and serological surveillance in livestock and humans should now be prioritized.

### High prevalence of *Coxiella burnetii* and implications for pathogen circulation

*Coxiella burnetii* is a globally distributed zoonotic pathogen responsible for Q fever, commonly associated with livestock and transmitted primarily through inhalation of contaminated aerosols, although tick-mediated transmission remains an important but underelucidated route in several ecosystems [82, 83]. In the present study, a remarkably high prevalence of *C. burnetii* (20.83%) was detected in questing *H. punctata* across multiple areas with ongoing enzootic transmission in Central and Northeastern Anatolia. This constitutes the first systematic demonstration of *C. burnetii* DNA in host-seeking *H. punctata* in Türkiye, expanding current knowledge beyond previous isolated findings from host-derived ticks.

The majority of positives originated from Central Anatolia, where *H. punctata* commonly infests cattle, sheep, and goats [4, 70], suggesting frequent contact with domestic reservoirs and a potentially overlooked role in pathogen circulation within pastoral environments. The detection of mixed infections—specifically co-occurrence with *B. major* and *T. orientalis*—further supports the capacity of *H. punctata* to acquire multiple TBPs simultaneously. Such co-harboring events, increasingly recognized as common in ticks, may influence pathogen–pathogen and pathogen–microbiome interactions—including those involving *C. burnetii*—and thereby affect co-transmission, transmission efficiency, and disease outcome in vertebrate hosts [84, 85]. Yet, these relationships remain largely unexplored.

Phylogenetic analyses of the *com1* and IS1111-Tnp loci revealed two closely related haplotypes circulating in Anatolia. Despite their low divergence, neither showed 100% identity to GenBank records, suggesting locally circulating—and potentially endemic—genotypes. Moreover, a notably clustered distribution was observed, with the majority of positives originating from the same locality, suggesting the presence of a cryptic but epidemiologically active area where *C. burnetii* circulation is likely ongoing. Historical data also demonstrate that *H. punctata* is capable of acquiring and maintaining *C. burnetii*. The earliest direct evidence dates back to Britain, where *C. burnetii* was successfully isolated from *H. punctata* collected from sheep, establishing this tick as a natural carrier of the pathogen [86]. Later, PCR-based surveys confirmed the presence of *C. burnetii* DNA in a questing *H. punctata* from Spain [87], as well as from Cyprus, where detections in ticks and their wildlife hosts suggested active sylvatic transmission cycles [52]. More recently, *H. punctata* has also been implicated in Mediterranean ecosystems as a potential contributor to local Q-fever maintenance, together with other sympatric ixodids [88], and similar findings from Xinjiang, China, where notable positivity rates were detected in *H. punctata* [61], indicate that this association is not geographically restricted but may extend across diverse Palearctic environments. Importantly, a similar geographical signal was observed in host-seeking *D. marginatus* collected in the same region, where a relatively high infection rate and frequent co-infections with SFG rickettsiae were documented [89], confirming the presence of a tick-associated *C. burnetii* transmission cycle, particularly in the northern part of Central Anatolia. Together with documented human seropositivity and Q-fever cases reported from nearby Bolu Province [90, 91], these data support that this area constitutes an active area with ongoing enzootic transmission of Q-fever in which ticks are epidemiologically involved. Although aerosol exposure remains the dominant transmission route for humans [82, 83], the detection of *C. burnetii* in questing *H. punctata*—as well as in questing *D. marginatus*—is consistent with the possibility of transstadial persistence, and suggests that *Dermacentor–Haemaphysalis* tick communities may play a contributory role in the local maintenance of the bacterium within enzootic cycles in Anatolia.

Given the high prevalence observed in host-seeking adults—independent of any bloodmeal source—the bacterium appears to be actively circulating in *H. punctata* populations. However, PCR positivity alone cannot confirm transmission competence. Determining whether *H. punctata* supports *C. burnetii* replication, transstadial or transovarial maintenance, or effective delivery to hosts will require controlled laboratory studies and vector-competence experiments. Still, the findings presented here strongly suggest that *H. punctata* should be prioritized among candidate vectors contributing to silent Q-fever dynamics in Anatolia. These observations further highlight the need for integrated One Health surveillance to clarify the relative contribution of ticks versus aerosol-based transmission in endemic areas and to better assess the risks posed to livestock and human populations.

### Spotted fever group rickettsiae: detection of *Rickettsia hoogstraalii* and *Candidatus* Rickettsia yenbekshikazakhensis

SFG rickettsiae are among the most commonly detected TBPs in tick specimens across Europe and parts of Asia, yet most records originate from host-derived ticks and the ecological role of questing individuals remains insufficiently understood [92]. In this study, three questing *H. punctata* from Northeastern Anatolia were PCR-positive for SFG rickettsiae. Sequence analyses showed that one tick carried *R. hoogstraalii*, confirmed through *gltA*-based BLAST identity with multiple reference sequences previously detected in *Haemaphysalis* ticks from Türkiye and other Mediterranean regions. Species identification for the remaining two positive ticks was based on combined sequence data from the *ompA* and *ompB* loci, both of which are informative markers for SFG rickettsiae [35, 92]. The *ompA* fragment showed its highest similarity to *R. aeschlimannii*-like sequences, a pattern best explained by the absence, at the time of analysis, of any reference *ompA* sequence for *Candidatus* R. yenbekshikazakhensis in GenBank rather than a true phylogenetic affinity. In contrast, *ompB* sequencing provided clear taxonomic resolution, showing 100% identity with *Candidatus* R. yenbekshikazakhensis previously reported from *H. punctata* in Kazakhstan. This confirms the presence of *Candidatus* R. yenbekshikazakhensis in natural *H. punctata* populations in Türkiye for the first time. Phylogenetically, the Anatolian strain clustered within the Kazakh *Candidatus* R. yenbekshikazakhensis clade with strong support, clearly separated from *R. aeschlimannii* and other SFG taxa. By adding new *ompA* sequence information, the present study therefore expands the available genetic reference dataset for this emerging lineage. Besides, the co-detection of *Candidatus* R. yenbekshikazakhensis and *B. major* in one tick further supports the role of *H. punctata* as a host for multiple microorganisms.

*Candidatus* R. yenbekshikazakhensis was first described from questing *H. punctata* in Kazakhstan, where it occurred at a high infection rate and was proposed as a tick-associated rickettsial lineage with potential epidemiological significance [60]. Additionally, this lineage has also recently been detected in small mammals from Kazakhstan [93]. The present finding from Anatolian host-seeking *H. punctata* therefore constitutes one of the earliest confirmations of this species beyond its original Kazakh focus, expanding its known biogeographical range westward across the Palearctic. Although no confirmed disease has yet been attributed to this species, its close phylogenetic proximity to pathogenic SFG rickettsiae (e.g., *R. aeschlimannii*), repeated detection in field-collected ticks, and apparent specialization for *H. punctata* highlight its potential epidemiological relevance. These features collectively underscore the need for in vitro isolation, experimental transmission studies, and serological surveys in livestock and humans to evaluate its veterinary and zoonotic significance.

*Rickettsia hoogstraalii* is a predominantly *Haemaphysalis*-associated SFG rickettsia originally described from *Haemaphysalis sulcata* in Croatia and later reported from multiple *Haemaphysalis* species across Eurasia [92, 94]. In Türkiye, this species has been repeatedly detected in *H. parva* and sporadically in *H. sulcata*, particularly in the central regions of Anatolia [54, 63, 95]. Its recovery here from a host-seeking *H. punctata* constitutes the first such record in Türkiye, expanding both the host range and ecological context of the species. Additional detections of *R. hoogstraalii* in *H. punctata* from other Mediterranean and southeastern European regions—including Cyprus, Sardinia, and Romania [58, 62, 96]—further indicate that this rickettsia is broadly associated with the *H. punctata* group across its Palearctic range. The repeated association of this lineage with several *Haemaphysalis* ticks may reflect an evolutionary specialization and point toward a stable long-term host–pathogen relationship. Although its pathogenic significance remains unknown, its phylogenetic proximity to zoonotic SFG rickettsiae, its frequent recovery from questing ticks, and its presence in regions with documented rickettsiosis risk warrant closer attention.

### Tick-borne piroplasmids: *Babesia* and *Theileria* in host-seeking *Haemaphysalis punctata*

Piroplasmid infections in *H. punctata* have historically received limited attention, largely owing to the scarcity of structured surveys targeting host-seeking individuals of this species. Nonetheless, early experimental and field studies demonstrated that *H. punctata* can act as a competent vector for *B. major* and *B. motasi* [13, 97–99]. Despite these observations, its broader and contemporary vector role across different geographic regions remains largely poorly understood. In the present study, *B. major* was detected in five questing ticks originating from ecologically distinct areas across both Central and Northeastern Anatolia, representing the most geographically extensive dataset for this pathogen in *H. punctata* to date. All five sequences clustered within *B. major*, yet each belonged to a unique haplotype, and none showed complete identity with GenBank records. This high haplotypic richness—despite the small number of positives—suggests previously unrecognized genetic diversity in *B. major* circulating within Anatolia. The observed relationships, including close similarity to sequences from China, Kyrgyzstan, and cattle from eastern Anatolia, indicate a complex biogeographic structure and possible host- or region-associated microevolution.

The detection of *B. major* in questing ticks is particularly significant, given previous reports from Türkiye, in which this species was documented only from host-derived *H. punctata* removed from humans [54]. Our findings therefore expand both the known geographic range and ecological context of *B. major* transmission. Mixed infections involving *B. major* and *Rickettsia*, *Coxiella*, or Burana virus additionally highlight the potential of *H. punctata* to harbor diverse pathogen assemblages simultaneously, a phenomenon with unclear but potentially important implications for vector competence, pathogen–pathogen interactions, and co-transmission dynamics. Although *B. major* has been reported from livestock in Türkiye [100], its epidemiological significance, reservoir host range, and local transmission dynamics remain insufficiently defined. The presence of *B. major* in host-seeking *H. punctata* across multiple areas with ongoing enzootic transmission suggests that the parasite circulates cryptically in the environment and underscores the need to clarify its ecology, clinical relevance, and potential impact on animal health.

In addition to *B. major*, *T. orientalis* was identified in one questing *H. punctata* from Central Anatolia. The haplotype exhibited complete identity with multiple sequences reported from southeastern Europe and Asia, including cattle-derived isolates from Bosnia and Türkiye, indicating that it belongs to a widely distributed lineage within the *T. orientalis* complex. Because the 18S rRNA gene does not reliably discriminate genotypes within the *T. orientalis* complex [101], the detected haplotype was conservatively assigned at the complex level. Members of the *T. orientalis* complex include both pathogenic and nonpathogenic genotypes [101], and therefore the clinical relevance of the detected lineage cannot be inferred from the present data. Its detection in *H. punctata* remains very rare, and the vector competence of this tick species for *T. orientalis* has never been formally evaluated. Experimental evidence for transstadial survival exists only from a single early study [102], and its relevance to contemporary *T. orientalis* epidemiology remains uncertain. In Türkiye, the *T. orientalis* complex circulates among cattle with multiple genetic lineages [103], and has also been detected in ticks collected from cattle—including *H. marginatum*, *D. marginatus*, *Ixodes ricinus*, *Alloceraea inermis*, and *H. parva*—as well as in a single host-seeking *I. ricinus* adult [63]. Despite these findings, the active tick vector(s) of the *T. orientalis* complex in Türkiye remain(s) undetermined, representing a crucial knowledge gap for animal health. Our detection in host-seeking *H. punctata* therefore broadens the pool of potential competent vectors and highlights this species as a priority target in future vector-competence studies.

Taken together, these findings indicate that piroplasmid circulation in *H. punctata* populations is likely underestimated, and that this species may play a more substantial role in natural maintenance cycles than currently recognized, thereby warranting targeted vector-competence investigations and broader ecological surveillance.

### Genetic diversity and phylogenetic position of the detected *Ehrlichia* sp.

In this study, an *Ehrlichia* genotype was identified from a single questing *H. punctata* in Central Anatolia. Although *Ehrlichia* spp. are well-recognized TBPs of veterinary and medical importance (e.g., *E. canis*, *E. chaffeensis*, and *E. ruminantium*) [104, 105], their diversity in *Haemaphysalis* ticks remains poorly characterized. The *groEL* sequence obtained here showed no identical matches in GenBank and exhibited its highest similarity (~ 96%) to an undescribed *Ehrlichia* lineage from *H. japonica* in Russia, suggesting a potentially novel or deeply divergent genotype. Phylogenetically, the sequence formed a distinct, well-supported branch within the *Ehrlichia* clade, clustering with Eurasian *Haemaphysalis*-associated genotypes from Russia and Central Africa. Reports of *Ehrlichia* in *H. punctata* are scarce but span diverse ecological contexts, including ticks collected from domestic animals [64, 106], wildlife [58], and even humans [107]. In Türkiye, an undescribed *Ehrlichia* genotype was previously detected in *H. punctata* collected from sheep [63], reinforcing this species as a potential carrier of poorly understood *Ehrlichia* lineages in pastoral ecosystems.

Together, these sporadic but ecologically diverse detections suggest that *H. punctata* repeatedly encounters and acquires *Ehrlichia* spp. in nature, even if most lineages remain molecularly unidentified. This pattern aligns with accumulating evidence that *Ehrlichia* diversity in *Haemaphysalis* ticks is substantially underrecognized, with molecular surveys in Eurasia revealing multiple deeply divergent lineages [108]. In Türkiye specifically, broad microorganism screening demonstrated the presence of numerous *Ehrlichia* genotypes in ticks associated with ruminants—including *H. punctata*, *H. parva*, *H. sulcata*, *Rhipicephalus turanicus*, *H. marginatum*, *Hyalomma excavatum*, *D. marginatus*, and *I. ricinus*—highlighting an underestimated diversity at the livestock–tick interface [63]. Although a single positive sample precludes any inference about vector competence, the detection of a genetically distinct lineage in a host-seeking individual—rather than a host-derived tick—indicates that cryptic *Ehrlichia* diversity is actively circulating within natural *H. punctata* populations in Anatolia. Future investigations incorporating multilocus sequencing, wider geographic sampling, and experimental transmission assays are needed to clarify the taxonomy, reservoir associations, and zoonotic potential of this lineage.

### Study limitations

This study has several limitations that should be acknowledged. First, although next-generation sequencing was successfully applied for full-genome characterization of Burana virus, comprehensive NGS-based analyses could not be performed for all detected microorganisms owing to technical complexity and financial constraints. Consequently, bacterial and protozoan agents were characterized using targeted multilocus PCR approaches rather than whole-genome data. Second, while the number of host-seeking *H. punctata* examined was sufficient to reveal previously undocumented pathogen circulation, the overall sample size remains moderate, and low-prevalence agents may therefore be underrepresented. Despite these limitations, the study provides robust molecular, phylogenetic, and ecological evidence for the circulation of diverse and, in some cases, previously unreported TBPs. Importantly, it establishes a strong foundation for future genomic, isolation-based, and vector-competence studies targeting *H. punctata* and its associated microbial community.

## Conclusions

This study provides the first comprehensive investigation of TBPs in host-seeking *H. punctata* from Türkiye and reveals a surprisingly rich and previously underappreciated microbial spectrum. The detection and full-genome characterization of Burana virus outside Central Asia significantly expand its known biogeographical range and demonstrate active circulation of this orthonairovirus within Anatolia. The high prevalence and localized clustering of *C. burnetii* suggest cryptic but epidemiologically active Q-fever activity in the region, consistent with a possible tick-associated maintenance cycle. The identification of both *R. hoogstraalii* and *Candidatus* R. yenbekshikazakhensis further emphasizes the strong affinity of *Haemaphysalis* ticks for SFG rickettsiae, while *B. major* and *T. orientalis* detections confirm ongoing piroplasmid circulation in local wildlife–livestock interfaces. Finally, the discovery of a genetically distinct *Ehrlichia* lineage in a questing tick underscores the hidden diversity of this genus in Eurasia. Collectively, these findings demonstrate that *H. punctata* plays a more substantial ecological role in the maintenance and dissemination of TBPs than previously recognized and highlight the necessity of targeted vector-competence studies, long-term surveillance, and pathogen isolation efforts to fully evaluate its impact on animal and human health.

## Supplementary Information

Supplementary Material 1.

Supplementary Material 2.

## Data Availability

The sequence data that support the findings of this study are openly available in GenBank (https://www.ncbi.nlm.nih.gov/genbank/) under accession numbers provided in Table 2. The geographical locations are provided in Table 1 and Table 2.
