# Supplementary material for: Molecular screening of tick-borne pathogens in host-seeking Haemaphysalis punctata Canestrini & Fanzago, 1878 (Ixodoidea: Ixodidae) in Anatolia with the first report of Burana virus
Source: Parasit Vectors. 2026 Mar 27;19:201. doi: 10.1186/s13071-026-07367-4 (PMC13147571; doi:10.1186/s13071-026-07367-4)
Supplement: Supplementary file 1 — Supplementary Material 1. [file 13071_2026_7367_MOESM1_ESM.docx]

**Figure S1.** Bayesian phylogenetic tree inferred from aligned nucleotide sequences of the 18S rRNA gene of piroplasms under the GTR+G4+I substitution model. *Cardiosporidium cionae* (EU052685) was used as the outgroup. The dataset comprised 53 sequences and 538 aligned positions. Node labels show posterior probabilities, with values <0.7 omitted for clarity. Haplotype sequences obtained in this study, and their corresponding clade, are highlighted in red. GenBank accession numbers are provided before species names. The clades corresponding to the two piroplasmid species detected in this study (*Babesia major* and *Theileria orientalis*), as well as the haplotypes generated here, are highlighted in red. The scale bar indicates the number of nucleotide substitutions per site.

**Figure S2.** Bayesian phylogenetic tree inferred from aligned nucleotide sequences of the *groEL* gene of *Ehrlichia* spp. under the TN93+G4 substitution model. *Neoehrlichia mikurensis* (AB074461) was used as the outgroup. The dataset comprised 37 sequences and 583 aligned positions. Posterior probabilities are displayed beside nodes, with values <0.75 omitted for clarity. The *Ehrlichia* haplotype detected in this study (Ehg-HpuHp1) and its corresponding clade are highlighted in red. GenBank accession numbers are provided before isolate names, and collection source information with country of origin for closely related isolates is shown after the names. The scale bar represents the number of nucleotide substitutions per site.

**Figure S3.** Bayesian phylogenetic tree generated from aligned nucleotide sequences of the *com1* gene of *Coxiella* spp., inferred using the HKY substitution model. *Candidatus* Coxiella mudrowiae (CP024961) was used as the outgroup. The dataset included 35 sequences with 449 aligned positions. Posterior probability values are shown at nodes, with values <0.80 omitted for clarity. The *Coxiella burnetii* haplotypes identified in this study (Cbc-HpuHp1 and Cbc-HpuHp2) and their corresponding cluster are highlighted in red. GenBank accession numbers precede isolate names, and host/source and country of origin follow each name. The scale bar indicates nucleotide substitutions per site.

**Figure S4.** Bayesian phylogenetic tree generated from aligned nucleotide sequences of the IS1111 element of *Coxiella* spp., inferred using the JC69 substitution model. *Coxiella* sp. (EU430257) was used as the outgroup. The dataset included 43 sequences with 529 aligned positions. Posterior probability values are shown at nodes, with values <0.75 omitted for clarity. The *Coxiella burnetii* haplotypes identified in this study (Cbi-HpuHp1 and Cbi-HpuHp2) and their corresponding cluster are highlighted in red. GenBank accession numbers precede isolate names, and host/source and country of origin follow each name. The scale bar indicates nucleotide substitutions per site.

**Figure S5.** Bayesian phylogenetic tree inferred from aligned nucleotide sequences of the *ompB* gene of *Rickettsia* spp., using the GTR+G4+I substitution model. *Rickettsia australis* (AF123709) was used as the outgroup. The dataset included 30 sequences with 778 aligned positions. Posterior probability values are shown at nodes; values <0.80 were removed for clarity. The *Candidatus* Rickettsia yenbekshikazakhensis haplotype identified in this study (Rye-HpuHp1) and its corresponding cluster are highlighted in red. GenBank accession numbers precede isolate names. The scale bar indicates the number of nucleotide substitutions per site.

**Figure S6.** Bayesian phylogenetic tree inferred from aligned nucleotide sequences of the *ompA* gene of *Rickettsia* spp., using the GTR+G4 substitution model. *Rickettsia australis* (AF149108) was used as the outgroup. The dataset included 28 sequences with 559 aligned positions. Posterior probability values are shown at nodes; values <0.75 were omitted for clarity. The *Candidatus* Rickettsia yenbekshikazakhensis haplotype detected in this study (Rye-HpuHp1) and its corresponding branch are highlighted in red. GenBank accession numbers are provided before isolate names. The scale bar indicates the number of nucleotide substitutions per site.
