# Supplementary material for: Molecular screening of tick-borne pathogens in host-seeking Haemaphysalis punctata Canestrini & Fanzago, 1878 (Ixodoidea: Ixodidae) in Anatolia with the first report of Burana virus
Source: Parasit Vectors. 2026 Mar 27;19:201. doi: 10.1186/s13071-026-07367-4 (PMC13147571; doi:10.1186/s13071-026-07367-4)
Supplement: Supplementary file 2 — Supplementary Material 2. [file 13071_2026_7367_MOESM2_ESM.docx]

**Supplementary Table 1.** Primer sets and PCR cycling conditions used for molecular detection of target organisms in *Haemaphysalis punctata*

| Target organism | Target gene  region | Type of PCR | Primers (F, R) | Product  size (bp) | Cycling parameters | Reference |
| --- | --- | --- | --- | --- | --- | --- |
| Tick | 16S rDNA | Conventional | 16S+1  16S-1 | ~460 | 94 °C 2 min, 35 cycles [94 °C 30 s, 56 °C 30 s, 72 °C 1 min], 72°C 10 min | Black and Piesman, 1994 |
| *Babesia* spp. | 18S rRNA | Conventional | BJ1  BN2 | 411-452 | 94 °C 2 min, 40 cycles [94 °C 30 s, 55 °C 30 s, 72 °C 1 min], 72°C 10 min | Casati et al.,  2006 |
| *Theileria* spp. | 18S rRNA | Nested | Thei F1 – Thei R1  Thei F2 – Thei R2 | ~1700  1417-1426 | 94 °C 2 min, 35 cycles [94 °C 30 s, 50 °C 30 s, 72 °C 2 min], 72°C 10 min (the first round)  94 °C 2 min, 35 cycles [94 °C 30 s, 52 °C 30 s, 72 °C 1 min:30 s] 72°C 10 min (the second round) | Heidarpour Bami et al., 2009 |
| *Hepatozoon* spp. | 18S rRNA | Conventional | HepF  HepR | ~666 | 94 °C 2 min, 40 cycles [94 °C 30 s, 55 °C 30 s, 72 °C 1 min], 72°C 10 min | Inokuma et al., 2002 |
| *Anaplasma*  *marginale*/*A. ovis* | *msp4* | Conventional | MSP45  MSP43 | 851 | 94 °C 2 min, 40 cycles [94 °C 30 s, 60 °C 30 s, 72 °C 1 min], 72°C 10 min | de la Fuente et  al., 2007 |
| *Anaplasma phagocytophilum* | *msp4* | Conventional | MAP4AP5  MSP4AP3 | 849 | 94 °C 2 min, 40 cycles [94 °C 30 s, 54 °C 30 s, 72 °C 1 min], 72°C 10 min | de la Fuente et  al., 2007 |
| *Anaplasma phagocytophilum/A. platys* | *groEL* | Semi-nested | EphplgroEL(569)F  EphplgroEL(1193)R  EphplgroEL(569)F  EphgroEL(1142)R | 624  573 | 94 °C 2 min, 35 cycles [94 °C 30 s, 54 °C 30 s, 72 °C 1 min], 72°C 10 min (the first round)  94 °C 2 min, 35 cycles [94 °C 30 s, 54 °C 30 s, 72 °C 1 min 72°C 10 min (the second round) | Alberti et  al., 2005 |
| *Rickettsia* spp. | *gltA* | Conventional | *Rp* CS.409d  *Rp* CS. 1258n | 750 | 94 °C 2 min, 40 cycles [94 °C 30 s, 54 °C 30 s, 72 °C 1 min], 72°C 10 min | Roux et al.,  1997 |
|  | *ompA* | Conventional | Rr. 190.70  Rr. 190.701 | 629-632 | 94 °C 2 min, 40 cycles [94 °C 30 s, 53 °C 30 s, 72 °C 1 min], 72°C 10 min | Fournier et al.,  1998 |
|  | *ompB* | Conventional | 120-2788  120-3599 | 816 | 94 °C 2 min, 40 cycles [94 °C 30 s, 50 °C 30 s, 72 °C 1 min], 72°C 10 min | Roux and Raoult, 2000 |
| *Borrelia burgdorferi* sensu lato | 5S-23S rRNA | Nested | RIS1 – RIS2  RIS3 – RIS4 | 226-266  ~200 | 94 °C 2 min, 35 cycles [94 °C 30 s, 52 °C 30 s, 72 °C 1 min], 72°C 10 min (the first round)  94 °C 2 min, 35 cycles [94 °C 30 s, 50 °C 30 s, 72 °C 1 min] 72°C 10 min (the second round) | Postic et al.,  1994; Sen *et al*., 2011 |
| *B. burgdorferi* sensu lato + *B. miyamotoi* | *fla* | Nested | 132f – 905r  220f – 823r | 774  604 | 94 °C 2 min, 35 cycles [94 °C 30 s, 50 °C 30 s, 72 °C 1 min], 72°C 10 min (the first round)  94 °C 2 min, 35 cycles [94 °C 30 s, 54 °C 30 s, 72 °C 1 min] 72°C 10 min (the second round) | Wodecka et al., 2010 |
| *Ehrlichia* spp. | 16S rRNA | Conventional | Ehr.u.for  Ehr.CCE.rev | 619 | 94 °C 2 min, 40 cycles [94 °C 30 s, 61 °C 30 s, 72 °C 1 min], 72°C 10 min | Engvall et al., 1996; Duscher et al., 2014 |
|  | *groEL* | Conventional | Ehr-*groEL*-F  Ehr-*groEL*-R | 590 | 94 °C 2 min, 40 cycles [94 °C 30 s, 50 °C 30 s, 72 °C 1 min], 72°C 10 min | Dahmani et al., 2017 |
| *Coxiella burnetii* | IS1111-Tnp | Nested | IS1111_F1 IS1111_R2  IS1111_F2  IS1111_R1 | 670  570 | 94 °C 2 min, 35 cycles [94 °C 30 s, 56 °C 30 s, 72 °C 1 min], 72°C 10 min (the first round)  94 °C 2 min, 35 cycles [94 °C 30 s, 56 °C 30 s, 72 °C 1 min] 72°C 10 min (the second round) | Duron 2015 |
|  | *com1* | Conventional | com1F  com1R | 470 | 94 °C 2 min, 40 cycles [94 °C 30 s, 56 °C 30 s, 72 °C 1 min], 72°C 10 min | Tokarevich et al., 2019 |
| *Francisella* spp. | 16S rRNA | Conventional | Fr153F0.1  Fr1281R0.1 | ~1170 | 94 °C 2 min, 40 cycles [94 °C 30 s, 60 °C 30 s, 72 °C 1 min], 72°C 10 min | Barns et al., 2005 |
|  | 17-kDA lipoprotein | Conventional | TUL4-435  TUL4-863 | 420 | 94 °C 2 min, 40 cycles [94 °C 30 s, 62 °C 30 s, 72 °C 1 min], 72°C 10 min | Sjöstedt et al., 1997 |
| *Orthonairovirus haemorrhagiae* (CCHFV) | S-segment | Nested | Eecf-F1  Eecf-R1  Eecf-F2  Eecf-R2 | 307  211 | 94 °C 2 min, 40 cycles [94 °C 30 s, 60 °C 30 s, 72 °C 1 min], 72°C 10 min  94 °C 2 min, 35 cycles [94 °C 30 s, 57 °C 30 s, 72 °C 1 min], 72°C 10 min | Midilli et al., 2009 |
| *Orthonairovirus*  *parahaemorrhagiae (*Aigai virus*)* | S-segment | Nested | Gre-F1  Gre-R1  Gre-F2  Gre-R2 | 593  469 | 94 °C 2 min, 40 cycles [94 °C 30 s, 57 °C 30 s, 72 °C 1 min], 72°C 10 min  94 °C 2 min, 35 cycles [94 °C 30 s, 57 °C 30 s, 72 °C 1 min], 72°C 10 min | Midilli et al., 2009 |
| Nairo-generic (*Orthonairovirus*) | L-segment | Touch-down | 6942  7385 | 446-463 | 94 °C 2 min, 5x9 cycles [94 °C 30 s, 42-50 °C 30 s, 72 °C 1 min], 72°C 10 min | Honig et al., 2004 |
